# Interaction of *Trypanosoma cruzi* Gp82 With Host Cell LAMP2 Induces Protein Kinase C Activation and Promotes Invasion

**DOI:** 10.3389/fcimb.2021.627888

**Published:** 2021-03-12

**Authors:** Thiago Souza Onofre, João Paulo Ferreira Rodrigues, Marina Tiemi Shio, Silene Macedo, Maria Aparecida Juliano, Nobuko Yoshida

**Affiliations:** ^1^ Departamento de Microbiologia, Imunologia e Parasitologia, Escola Paulista de Medicina, Universidade Federal de São Paulo, São Paulo, Brazil; ^2^ Departamento de Biofísica, Escola Paulista de Medicina, Universidade Federal de São Paulo, São Paulo, Brazil

**Keywords:** *Trypanosoma cruzi*, metacyclic trypomastigote, host cell invasion, gp82, LAMP2, protein kinase C

## Abstract

The surface molecule gp82 of metacyclic trypomastigote (MT) forms of *Trypanosoma cruzi*, the protozoan parasite that causes Chagas disease, mediates the host cell invasion, a process critical for the establishment of infection. Gp82 is known to bind to the target cell in a receptor-dependent manner, triggering Ca^2+^ signal, actin cytoskeleton rearrangement and lysosome spreading. The host cell receptor for gp82 was recently identified as LAMP2, the major lysosome membrane-associated protein. To further clarify the mechanisms of MT invasion, we aimed in this study at identifying the LAMP2 domain that interacts with gp82 and investigated whether target cell PKC and ERK1/2, previously suggested to be implicated in MT invasion, are activated by gp82. Interaction of MT, or the recombinant gp82 (r-gp82), with human epithelial HeLa cells induced the activation of Ca^2+^-dependent PKC and ERK1/2. The LAMP2 sequence predicted to bind gp82 was mapped and the synthetic peptide based on that sequence inhibited MT invasion, impaired the binding of r-gp82 to HeLa cells, and blocked the PKC and ERK1/2 activation induced by r-gp82. Treatment of HeLa cells with specific inhibitor of focal adhesion kinase resulted in inhibition of r-gp82-induced PKC and ERK1/2 activation, as well as in alteration of the actin cytoskeleton architecture. PKC activation by r-gp82 was also impaired by treatment of HeLa cells with inhibitor of phospholipase C, which mediates the production of diacylglycerol, which activates PKC, and inositol 1,4,5-triphosphate that releases Ca^2+^ from intracellular stores. Taken together, our results indicate that recognition of MT gp82 by LAMP2 induces in the host cell the activation of phosholipase C, with generation of products that contribute for PKC activation and the downstream ERK1/2. This chain of events leads to the actin cytoskeleton disruption and lysosome spreading, promoting MT internalization.

## Introduction

The major lysosome-associated membrane glycoproteins LAMP1 and LAMP2 are heavily glycosylated proteins, contain a single membrane-spanning segment, a major portion that resides in the luminal side of lysosomes and a short cytosolic tail (Fukuda et al., 1988; Howe et al., 1988; Granger et al., 1990) Their extensive glycosylation is apparently not necessary for normal targeting, stability, or lysosome function (Kornfeld and Mellman, 1989). Despite the strong homology, LAMP1 and LAMP2 are distinct molecules, encoded by separate genes on different chromosomes (Mattei et al., 1990). Comparison of known *lamp* sequences among different species has shown that human LAMP1 has more similarity to LAMP1 from other species than to human LAMP2, and this also applies to LAMP2 (Fukuda et al., 1988). LAMP proteins have been detected on the plasma membrane of human cell lines and their expression was shown to increase after exposure to a lysosomotropic reagent (Mane et al., 1989). LAMP1 and LAMP2 may have different functions. It has been shown, for instance, that surface LAMP1, but not LAMP2, protects natural killer cells from degranulation-associated damage (Cohnen et al., 2013) and that LAMP2, but not LAMP1, plays a critical role in endosomal cholesterol transport (Schneede et al., 2011).

Lysosomes play an important role in host cell invasion by *Trypanosoma cruzi*, the protozoan parasite that causes Chagas disease. Interaction of *T. cruzi* with mammalian cell induces the exocytosis of lysosomes, which contributes for the parasitophorous vacuole formation (Tardieux et al., 1992; Rodríguez et al., 1995; Martins et al., 2011). Using different infective forms, namely metacyclic trypomastigote (MT) and tissue culture-derived trypomastigote (TCT), which correspond respectively to the insect-borne and mammalian host bloodstream parasites, the involvement of LAMP proteins in *T. cruzi* invasion has been investigated. Studies with TCT have implicated either LAMP1 or LAMP2. Cells with increased expression of LAMP1 at the surface were found to be more susceptible to invasion by TCT, the LAMP1 cytoplasmic tail motif, and not the surface-exposed luminal domain, playing the role of modulating the parasite entry (Kima et al., 2000). More recently, it was reported that LAMP2 plays a major role in TCT invasion, by influencing the distribution of caveolin-1 at the cell plasma membrane, which is crucial for plasma membrane repair (Couto et al., 2017). TCT is internalized in a vacuole expressing plasma membrane markers (Woolsey et al., 2003) and the internalization mimics a process of plasma membrane injury and repair that involves exocytosis of lysosomes (Fernandes et al., 2011). MT is internalized in a vacuole expressing lysosome markers (Martins et al., 2011; Cortez et al., 2016), requires LAMP2, but not LAMP1, and does not rely on the plasma membrane repair mechanism (Rodrigues et al., 2019).

Host cell invasion by MT is mediated by the stage-specific surface molecule gp82 (Yoshida, 2006). Gp82 binds to target cells in a receptor-mediated manner and induces the lysosome mobilization to the cell periphery that culminates in exocytosis (Martins et al., 2011; Cortez et al., 2016). There are indications that gp82-mediated MT binding triggers the target cell signaling cascade involving protein kinase C (PKC) and the extracellular signal-regulated protein kinases (ERK1/2) (Martins et al., 2011; Onofre et al., 2019). Recently, LAMP2 was identified as the host cell receptor for gp82 (Rodrigues et al., 2019). In this study we aimed at identifying the LAMP2 domain that interacts with gp82 and investigated whether target cell PKC and ERK1/2 are activated by gp82.

## Materials and Methods

### Modeling of Gp82 and LAMP2 for Protein–Protein Interaction Analysis

The amino acid sequences of gp82 (GenBank accession number L14824) and LAMP2 (UniProtKB P13473) were used to predict the protein models. Residues 1-29 (N-terminal signal peptide) and 500-516 (a nonpolar region at the extreme C-terminus) of gp82 were excluded after its identification, using PSORT II Prediction (https://psort.hgc.jp/) and PrediSI (http://www.predisi.de/) for signal peptide and TMHMM Server v.2.0 (https://services.healthtech.dtu.dk/service.php?TMHMM-2.0) for C-terminus region. As regards LAMP2, amino acids 1-28 (N-terminal signal peptide) and 378-410 (transmembrane and cytoplasmic regions) were excluded, as described on the page where the sequence was obtained and confirmed by the same on-line tools used for gp82. 3D model of gp82 was generated in on-line server Phyre² (Protein Homology/analogY Recognition Engine V 2.0) (Kelley et al., 2015), using intensive modelling mode, and that of LAMP2 was generated in SWISS-MODEL Interactive Workspace (Waterhouse et al., 2018), without template. Both models were submitted in YASARA Energy Minimization Server (Krieger et al., 2009) and were checked using: RAMPAGE (Ramachandran Plot Assessment) (Lovell et al., 2003), ProSA-web (Protein Structure Analysis) (Sippl, 1993; Wiederstein and Sippl, 2007), PROCHECK (Laskowski et al., 1993; Laskowski et al., 1996), ERRAT (Colovos and Yeates, 1993) and Verify 3D (Bowie et al., 1991; Lüthy et al., 1992). Afterwards, the analysis of protein-protein docking was made in on-line server ClusPro (https://cluspro.org), which provides a simple home page for basic use, requiring only two files in Protein Data Bank format (Kozakov et al., 2013; Kozakov et al., 2017; Vajda et al., 2017). All models and protein-protein docking were visualized in PyMOL Molecular Graphics System, Version 2.1.1 Schrödinger, LLC.

### Parasites, Mammalian Cells, and Cell Invasion Assay

*T*. *cruzi* strain CL was used throughout this study. The parasites were maintained alternately in mice and in liver infusion tryptose medium containing 5% fetal bovine serum. To stimulate differentiation of epimastigotes into metacyclic forms, the parasites were cultivated for one passage in Grace’s medium (Life Technologies/Thermo Fisher Scientific). Metacyclic forms were purified in a DEAE-cellulose column as described (Teixeira and Yoshida, 1986). Human epithelial HeLa cells were maintained in RPMI medium supplemented with 10% bovine fetal serum and invasion assays were performed according to the procedure described elsewhere (Rodrigues et al., 2017), by incubating the cells with MT at MOI = 10. A total of 250 Giemsa-stained cells was counted to quantify internalized MT.

### Membrane Fractionation

HeLa cells grown in 150 mm^2^ dishes (1x10^7^ cells per dish) were washed with PBS, and once with a buffer solution containing 250 mM glucose, 50 mM Tris, 5 mM MgCl_2_, pH 7.0. After scraping, the cells were sonicated, at power of 40% (Active Motif’s EpiShearTM sonication systems – Probe 3.2 mm), for three cycles of 5 sec and rest of 30 sec. The supernatant, obtained by centrifugation of 250 x g for 30 min, was further centrifuged at 100,000 x g for 1 h. The supernatant containing cytosolic fraction was collected and the pellet, containing membrane fraction, was washed and resuspended in buffer solution containing 1x protease cocktail inhibitor (Roche), 2 mM Na_3_VO_4_, 1 mM NaF and 1% Triton x-100.

### Detection of Phosphorylated Protein Kinase C and ERK1/2 by Western Blotting

HeLa cells were washed with PBS and lysed with a solution containing 10 mM Tris pH 7.5, 1 mM EDTA, 100 mM NaCl, 1% Igepal, 10% glycerol, protease cocktail inhibitor, 2 mM Na_3_VO_4_ and 1 mM NaF. Equal amounts of detergent soluble supernatant or cytosolic/membrane fractions were subjected to 10% acrylamide gel. After transfer onto nitrocellulose or PVDF membrane, they were subjected to immunoblot analysis with the first antibody diluted in TBS-T (50 mM Tris-HCl, pH 7.5, 150 mM NaCl and 0.1% Tween 20) plus 5% BSA, followed by washings in the same solution without BSA and incubation with the appropriate HRP-conjugated secondary antibody. In some experiments, the membranes were stripped from antibodies, using the following protocol. The membranes were soaked in 20 ml stripping buffer (0.2% SDS, 62 mM Tris-HCl pH 6.8, 160 µl β-mercaptoethanol) for 15 min at 56°C. For relative quantification of protein bands in western blot films, we used GelAnalyzer 19.1 software, in which the density of each band was converted to peaks and the area under the peak was used to calculate pixel volume.

### Production of Recombinant Gp82 Protein and Cell Binding Assay

The recombinant protein coded by the full-length *T. cruzi* gp82 sequence (GenBank accession number L14824), in frame with glutathione S-transferase (GST), was produced and purified as detailed (Cortez et al., 2006). As the expression of recombinant gp82 protein (r-gp82) in *Escherichia coli* leads to the formation of inclusion bodies, the purification was carried out by excision of the corresponding band (~75 kDa) from SDS-PAGE gel. This precludes contamination with LPS, provided that LPS from different bacteria, including *E. coli*, are detected as bands ranging 3.7-4.5 kDa in SDS-PAGE gel (Amano et al., 1988). We did not detect bands corresponding to bacterial components in r-gp82 preparation when it was run in SDS-PAGE gel, side by side with *E. coli* extract, and was subjected to western blotting, using anti-gp82 monoclonal antibody and anti-*E. coli* antiserum (**Figure S1**). For cell binding assay, HeLa cells were seeded onto 96-well microtiter plates at 4x10^4^ cells/well and were grown overnight at 37°C. After fixation with 4% paraformaldehyde in PBS, washings with PBS and blocking with PBS containing 2 mg/ml BSA (PBS-BSA) for 1 h at room temperature, the cells were incubated for 1 h at 37°C with r-gp82 in PBS-BSA. Following washes in PBS containing 0.1% Tween 20 and 1 h incubation with anti-gp82 polyclonal antiserum diluted in PBS-BSA, the cells were incubated with anti-mouse IgG conjugated to peroxidase. The bound enzyme was revealed using *o*-phenylenediamine and the absorbance at 490 nm was read in ELx800™ microplate reader (BioTek).

### Indirect Immunofluorescence Assay for Visualization of Lysosomes and Actin Cytoskeleton

For microscopy visualization of lysosomes and F-actin, HeLa cells were processed essentially as previously described (Onofre et al., 2019). Alexa Fluor 488 phalloidin or TRITC-phalloidin and DAPI were used to detect F-actin and DNA, respectively. For lysosome visualization, anti-human LAMP2 antibody and Alexa Fluor 488-conjugated anti-mouse IgG were used. After mounting the coverslips with adherent cells in ProLong Gold (Invitrogen), confocal images were acquired in a confocal microscope (Instituto de Farmacologia e Biologia Molecular (INFAR), Universidade Federal de São Paulo), using 63X objective, and were processed/analyzed using Leica LAS AF (Leica, 2012, Germany) and Imaris (Bitplane) software. The relative position of lysosomes in confocal images was evaluated by ImageJ 1.53c software. Cells were selected, and then green pixels (lysosomes) and blue pixels (nucleus) were plotted in a histogram. In some experiments, images were acquired in Olympus fluorescence microscope BX51 coupled to a Olympus DP71 camera, using Image-Pro Plus software.

### Treatment of HeLa Cells

In assays in which HeLa cells were incubated with synthetic peptide or drugs, which were dissolved in DMSO, the same amount of vehicle was present in untreated controls.

### Antibodies and Reagents

Antibodies directed to phospho-PKC α/βII (Thr638/641), phospho-PKC (pan) (γThr514), phospho-p44/42MAPK (ERK1/2) (Thr202/Tyr204), m-TOR, PTEN, β-Tubulin and glyceraldehyde-3-phosphate dehydrogenase (GAPDH) were from Cell Signaling Technology. Anti-LAMP2 (H4B4) antibody was from Developmental Studies Hybridoma Bank developed under the auspices of the NICHD and maintained by The University of Iowa, Department of Biology, Iowa City, IA 52242. Alexa Fluor 488 phalloidin or TRITC-phalloidin and Alexa Fluor 488-conjugated anti-mouse IgG were from Thermo Fisher Scientific. FAK inhibitor PF573228, PLC inhibitor U73122 and PKC activator PMA were from Sigma/Merck.

### Statistical Analysis

The Student’s *t* test, as implemented in GraphPad Prism software (Version 6.01), was used.

## Results

### Interaction of *Trypanosoma cruzi* Gp82 and Host Cell LAMP2 Is Inferred From the Structural Models

**Figure 1:**
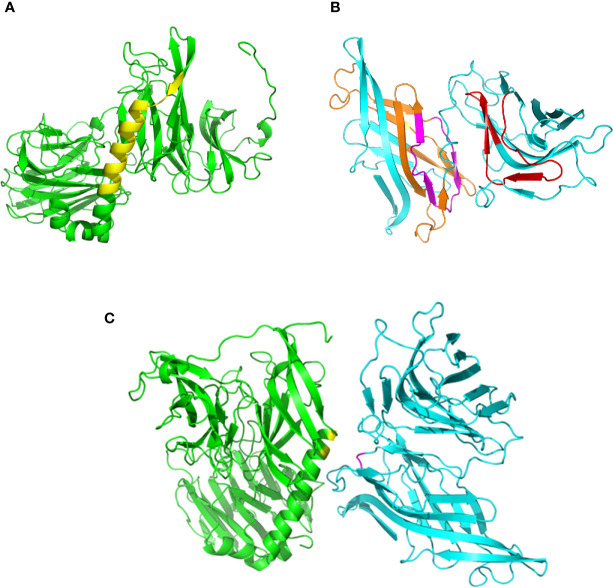
Structural models of gp82 and LAMP2 proteins and their presumed interaction. **(A)** *T. cruzi* surface molecule gp82, without residues 1–29 (N-terminus) and 500-516 (C-terminus). The cell binding site is highlighted (yellow). **(B)** LAMP2 without residues 1–29 (N-terminus) and 378–410 (C-terminus). Domains that potentially interact with gp82 are indicated (orange and red). Also indicated is the presumed site of interaction with gp82 cell binding site (magenta). **(C)** Highlighted in the model of gp82–LAMP2 interaction are the gp82 residues E259 and K262 (yellow) and the presumed LAMP2 amino acid residues involved in the interaction (magenta).

Using on-line servers Phyre^2^ and Swiss-model, we generated 3D models of gp82, without the residues 1-29 at the N-terminus and 500-516 at the C-terminus (**Figure 1A**), and of LAMP2 without residues 1-29 and 378-410 (**Figure 1B**). According to a previous finding, the main host cell binding site of gp82, corresponding to the sequence LARLTEELKTIKSVLSTWSK (Manque et al., 2000), is part of an α-helix that connects the N-terminal β-propeller domain to the C-terminal β-sandwich domain (Cortez et al., 2012). We searched for the LAMP2 domain that could interact with the referred gp82 sequence at the distance of up to 4 Å, taking into account that it contains the acidic residues E259 and E260, critical for host cell binding, in addition to basic residues K262 and K265 (Manque et al., 2000; Cortez et al., 2012). Two LAMP2 regions that potentially interact with gp82 were found, and in one of them nested the presumed site for gp82 binding (**Figure 1B**). Shown in **Figure 1C** is one of the predicted models of gp82-LAMP2 docking, in which the gp82 residues E259/K262 and the presumed LAMP2 amino acid residues N148/D149 involved in the interaction are highlighted.

### Peptide P5 Based on LAMP2 Sequence Inhibits Gp82 Binding to Host Cells

**Figure 2:**
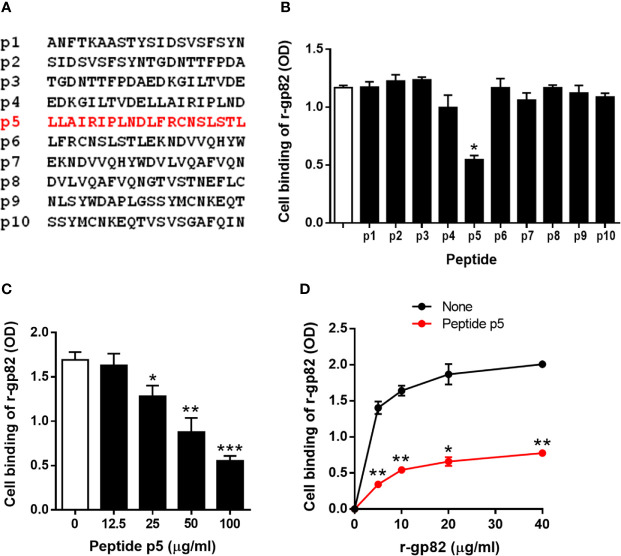
Inhibition of gp82 binding to cells by peptide p5. **(A)** Sequences of peptides spanning the LAMP2 domain identified as the site of interaction with gp82. **(B)** HeLa cells were incubated with r-gp82, in absence or in the presence of the indicated peptide. Binding was measured by ELISA. Representative results of one of three assays are shown. Values are the means ± SD of triplicates. Inhibition by peptide p5 was significant (*P < 0.0001). **(C)** HeLa cells were incubated with r-gp82, at 40 μg/ml, in absence or in the presence of peptide p5 at the indicated concentrations. Values are the means ± SD of three experiments performed in triplicate. Inhibition by peptide p5 at different concentrations was significant (*P < 0.01, **P < 0.005, ***P < 0.0001). **(D)** HeLa cells were incubated with r-gp82, at the indicated concentrations, in absence or in the presence of peptide p5 at 100 µg/ml. Values are the means ± SD of triplicates from a representative assay. Binding of r-gp82 to cells was significantly inhibited in the presence of peptide p5 (*P < 0.0005, **P < 0.0001).

To determine the LAMP2 sequence involved in gp82 binding, we used synthetic 20-mer peptides, spanning the region presumed to be the domain that interacts with gp82 (**Figure 2A**). Out of ten, eight peptides (p1-p8) had an overlapping of 10 residues. There was no overlapping between peptides p8 and p9, which were separated by 127 residues that did not attain the proximity of 4 Å for gp82 interaction. The peptides were tested for the ability to inhibit gp82 binding to host cells. We used the recombinant gp82 protein (r-gp82), which was shown previously to have a host cell binding capacity comparable to that of the native gp82 (Ruiz et al., 1998). GST, to which r-gp82 is fused, is devoid of ability to bind to HeLa cells (Cortez et al., 2006; Ferreira et al., 2006; Zanforlin et al., 2013; Martins et al., 2015). Microtiter plates coated with HeLa cells were incubated with the r-gp82, at 40 µg/ml, in absence or in the presence of individual peptides, at 200 µg/ml. Binding of r-gp82 to cells was significantly inhibited by peptide p5 (**Figure 2B**). Next, the effect of varying concentrations of peptide p5 was determined. Peptide p5 inhibited r-gp82 binding to cells in a dose-dependent manner (**Figure 2C**). An assay was also performed in which HeLa cells were incubated with r-gp82 at varying concentrations, in absence or in the presence of peptide p5 at 100 µg/ml. At all concentrations, r-gp82 bound significantly less to HeLa cells in the presence of peptide p5 (**Figure 2D**).

### Gp82-Mediated Metacyclic Trypomastigote Invasion of Host Cells Is Inhibited by Peptide P5

**Figure 3:**
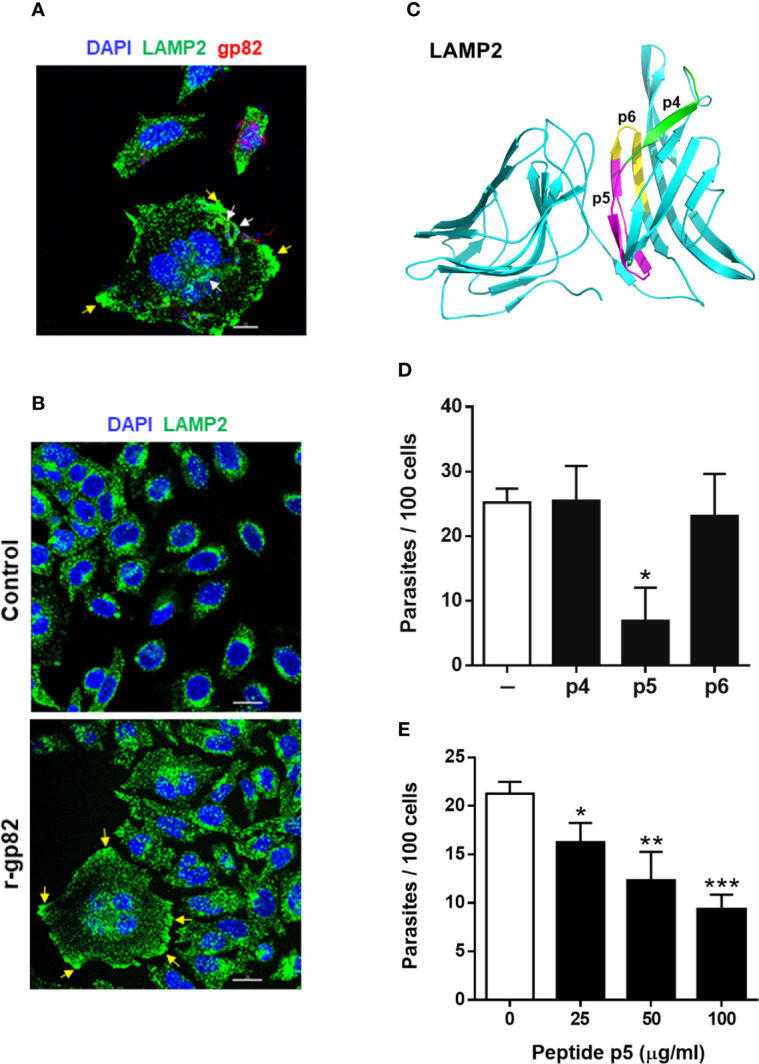
Inhibition of gp82-mediated MT invasion of host cells by peptide p5. **(A)** HeLa cells were incubated with MT for 30 min and then processed for confocal fluorescence microscopy to visualize lysosomes (green), nucleus (blue), and adherent parasites (red). Scale bar = 10 µm. Note the internalized MT with lysosome marker (white arrows) and lysosome accumulation at the cell edges (yellow arrows) in a multinucleated large cell. **(B)** HeLa cells were incubated for 30 min in absence or in the presence of recombinant gp82 (r-gp82) and visualized by confocal microscopy. Scale bar = 20 µm Note the perinuclear localization of lysosomes in control cells, the lysosome spreading in cells incubated with r-gp82 and the accumulation at edges (yellow arrows) in a multinucleated cell. **(C)** The 3D model of LAMP2, highlighting the peptide p5 sequence (magenta), the sequences of p4 (green) and p6 (yellow) that do not overlap with p5. **(D)** HeLa cells were incubated for 1 h with MT in absence or in the presence of the indicated peptide, at 100 μg/ml, and processed for intracellular parasite quantification. Values are the means ± SD of four independent assays performed in duplicate. MT invasion was significantly reduced in the presence of peptide p5 (*P <0.001). **(E)** HeLa cells were incubated for 1 h with MT in absence or in the presence of peptide p5 at the indicated concentrations, and the internalized parasites was quantified. Values are the means ± SD of three independent assays performed in duplicate. Inhibition of MT internalization by peptide p5 was significant at all concentrations (*P < 0.05, **P <0.01, ***P < 0.0005).

We examined the ability of peptide p5 to interfere with gp82-mediated MT internalization. In the invasion process, we have consistently seen that after 1 h incubation of HeLa cells with MT, at MOI=10, approximately 25% of cells were invaded (**Figure S2A**), most of them harboring one parasite per cell (**Figure S2B**). Large cells, with more than one nucleus, were apparently more susceptible to MT invasion, harboring a few parasites per cell (**Figure S2C**). The average number of cells with more than one nucleus was not higher than 10%, and those that were infected represented about 50%. Metacyclic forms are internalized in a vacuole expressing lysosome membrane markers (Cortez et al., 2016; Rodrigues et al., 2019), as shown in **Figure 3A**. In large multinucleated cells, a few parasites per cell could be seen in a lysosome membrane-derived vacuole, upon reaction with anti-LAMP2 antibody (**Figures 3A** and **S3**). Clearly evident in these cells is the spreading of lysosomes and accumulation at the edges (**Figures 3A** and **S3**), a profile that is also observed in cells incubated with r-gp82 protein (**Figure 3B**). The relative position of lysosomes was quantified in selected cells (11 from control and 13 from samples treated with r-gp82), by plotting green pixels (lysosomes) and blue pixels (nucleus) in a histogram. Uninucleated as well as multinucleated cells were included in the analysis. In the histogram plotted to compare quantitatively the lysosomes positioned away from the nucleus, a higher number could be seen in cells incubated with r-gp82 than in control cells (**Figure S4**).

To test the effect of peptide p5 on MT invasion, peptides p4 and p6 were used as controls. In the 3D model of LAMP2, the sequences corresponding to these peptides are located on the surface **(** **Figure 3C**). HeLa cells were incubated for 1 h with MT, in absence or in the presence of peptide p4, p5 or p6, at 100 µg/ml, and were processed for intracellular parasite quantification. Peptide p5 significantly inhibited MT internalization whereas peptides p4 and p6 had no inhibitory effect (**Figure 3D**). Next, the effect of varying concentrations of peptide p5 was determined. Peptide p5 inhibited MT invasion in a dose-dependent manner (**Figure 3E**).

### Host Cell Protein Kinase C and ERK1/2 Are Activated Upon Interaction With Metacyclic Trypomastigote

**Figure 4:**
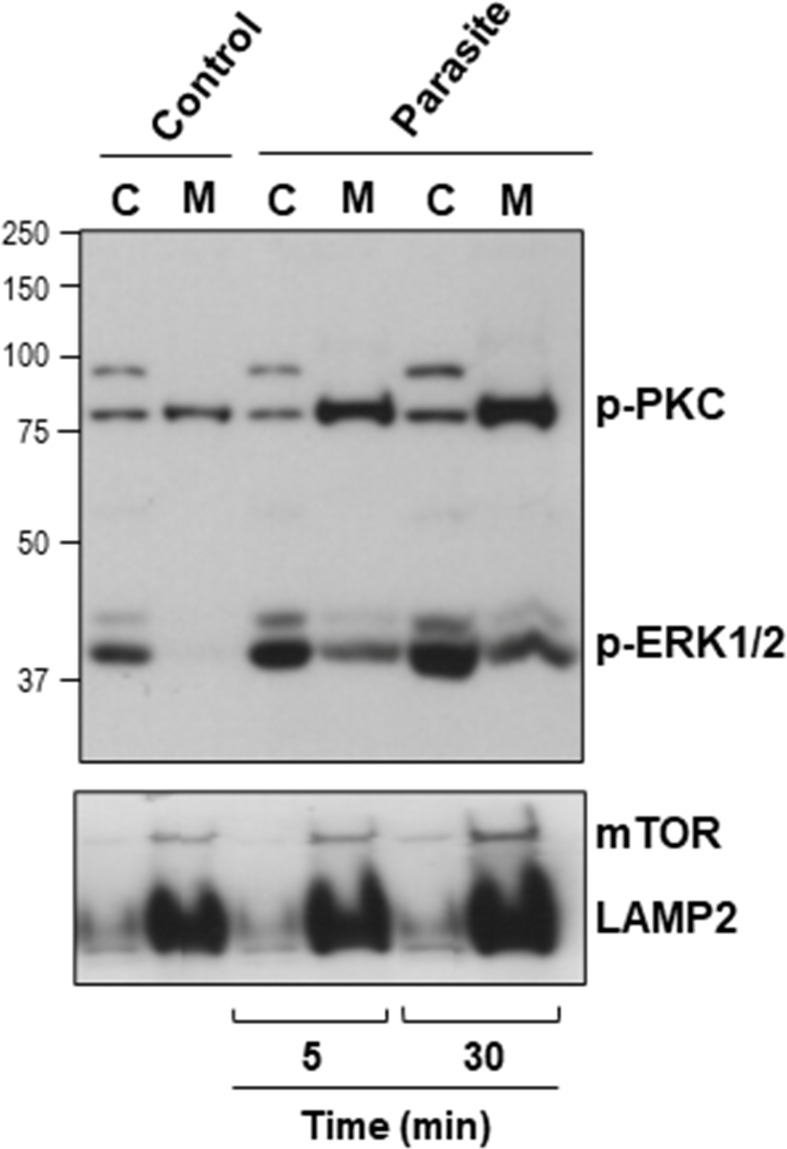
Activation of host cell PKC and ERK1/2 upon interaction with MT. HeLa cells were incubated in absence or in the presence of MT for the indicated time. Cytosolic (C) and membrane (M) fractions were isolated and analyzed using antibody to phospho-PKC and to phospho-ERK1/2. Anti-LAMP2 and anti-mTOR antibodies were used as fractionation control. Note the increase in the phosphorylation levels of PKC and ERK1/2 induced by MT.

The host cell PKC and ERK1/2 have been implicated in gp82-mediated MT internalization (Martins et al., 2011; Onofre et al., 2019). To examine whether MT effectively induced the activation of these kinases, HeLa cells were incubated with MT for 5 or 30 min and then processed for western blotting, along with the control cells that had no contact with parasites. As active PKC translocates to the plasma membrane, we isolated membrane and cytosol fractions, which were analyzed by western blotting using antibody to phospho-PKCα/βII and to phospho-ERK1/2. As control for the correct fractionation, antibody to LAMP2 was used. Detection of mammalian target of rapamycin (mTOR) was included in this assay, because there are indications that MT associates with mTOR and LAMP2 at peripheral lyososomes (Cortez et al., 2016). The increase in the phosphorylation levels of PKC and ERK1/2 was detectable after 5 min interaction of HeLa cells with MT, and was more pronounced after 30 min (**Figure 4**). Activated PKC was detected predominantly in the membrane fraction, whereas activated ERK1/2 remained mostly in the cytosolic fraction (**Figure 4**). LAMP2 and mTOR partitioned in the membrane fraction and the higher intensity in cells incubated with MT for 30 min (**Figure 4**) is possibly due to lysosome biogenesis induced by gp82, as previously observed (Cortez et al., 2016).

### Gp82-Induced Activation of Host Cell Protein Kinase C and ERK1/2 Is Blocked by Peptide P5

**Figure 5:**
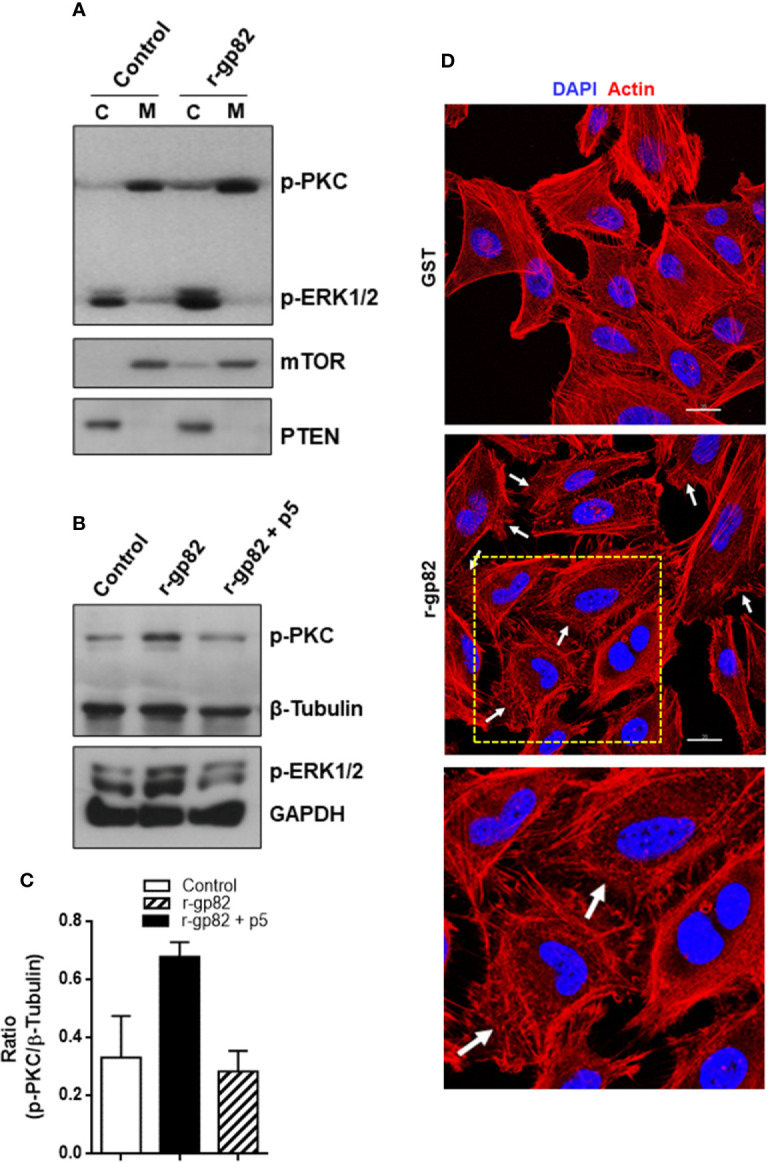
Blockage of gp82-induced activation of host cell PKC and ERK1/2 by peptide p5. **(A)** HeLa cells were incubated for 30 min in absence or in the presence of r-gp82. The cytosolic (C) and membrane (M) fractions were analyzed by Western blotting using antibody to phospho-PKC and to phospho-ERK1/2. Note the increased phosphorylation levels of PKC and ERK1/2 upon incubation with r-gp82, and the detection of PKC and ERK1/2 predominantly in the membrane and cytosolic fractions, respectively. **(B)** HeLa cells were incubated for 30 min with r-gp82 in absence or in the presence of peptide p5. The western blot was revealed with antibodies directed to: phospho-PKC, phospho-ERK1/2, β-tubulin or GAPDH. Note the impairment of gp82-induced PKC and ERK1/2 activation by peptide p5. **(C)** Densitometry analysis of Western blots were performed. Values are the means ± SD of three independent assays. **(D)** HeLa cells were incubated for 30 min in absence or in the presence of r-gp82 and then processed for visualization of actin cytoskeleton (red) and nucleus (blue). Scale bar = 20 µm. Note the disrupted cortical F-actin in cells incubated with r-gp82 (white arrows). To facilitate visualization, a magnified image from the framed area (square) is also shown.

First, we determined the effect of gp82 on PKC and ERK1/2 activation, HeLa cells were incubated for 30 min in absence or in the presence of r-gp82 at 10 µg/ml, the membrane and cytosolic fractions were isolated and analyzed by western blotting. The phosphorylation levels of PKC and ERK1/2 increased upon interaction with r-gp82, PKC being detected predominantly in the membrane fraction and ERK1/2 in the cytosolic fraction (**Figure 5A**). The membrane fraction was enriched in mTOR and the cytosolic fraction in phosphatase PTEN (**Figure 5A**). To demonstrate that PKC activation is induced by gp82-mediated interaction of MT with host cells, an additional experiment was performed. The parasites were incubated in absence or in the presence of monoclonal antibody directed to gp82 for 30 min and then were seeded onto HeLa cells. After 30 min incubation, the cells that interacted with MT and the control cells that had no contact with parasites were processed for western blotting and detection of phosphorylated PKC. Anti-gp82 monoclonal antibody reduced the capacity of MT in activating PKC (**Figure S5**). Treatment of HeLa cells with anti-gp82 monoclonal antibody did not have any effect.

Next, the effect of peptide p5 on gp82-induced PKC and ERK1/2 activation was determined. HeLa cells were incubated for 30 min with r-gp82 at 10 µg/ml, in absence or in the presence of peptide p5 at 50 µg/ml. The recombinant gp82 and peptide p5 were absent in control cells. After western blotting of cell extracts, the nitrocellulose membranes were revealed with antibody directed to phosphoylated PKC and ERK1/2, as well as β-tubulin or GAPDH, which served as loading controls. Both PKC and ERK1/2 had their phosphorylation levels increased upon interaction with r-gp82, an effect that was counteracted by peptide p5 (**Figure 5B**). Quantification of western blot bands using GelAnalyzer 19.1 software confirmed the increase in PKC activation induced by r-gp82 and inhibition by peptide p5 in repeated assays (**Figure 5C**). PKC and ERK1/2 have been associated with actin cytoskeleton organization in different cell types (Nurminsky et al., 2007; Wang and Hatton, 2007). F-actin rearrangement is induced by r-gp82 (Cortez et al., 2006), what we confirmed by incubating HeLa cells for 30 min with r-gp82 at 20 µg/ml and then processing for visualization at the confocal microscope. Disruption of F-actin was detectable in cells incubated with r-gp82 (**Figure 5D**).

### Focal Adhesion Kinase Inhibitor Affects Gp82-Induced Protein Kinase C Activation

**Figure 6:**
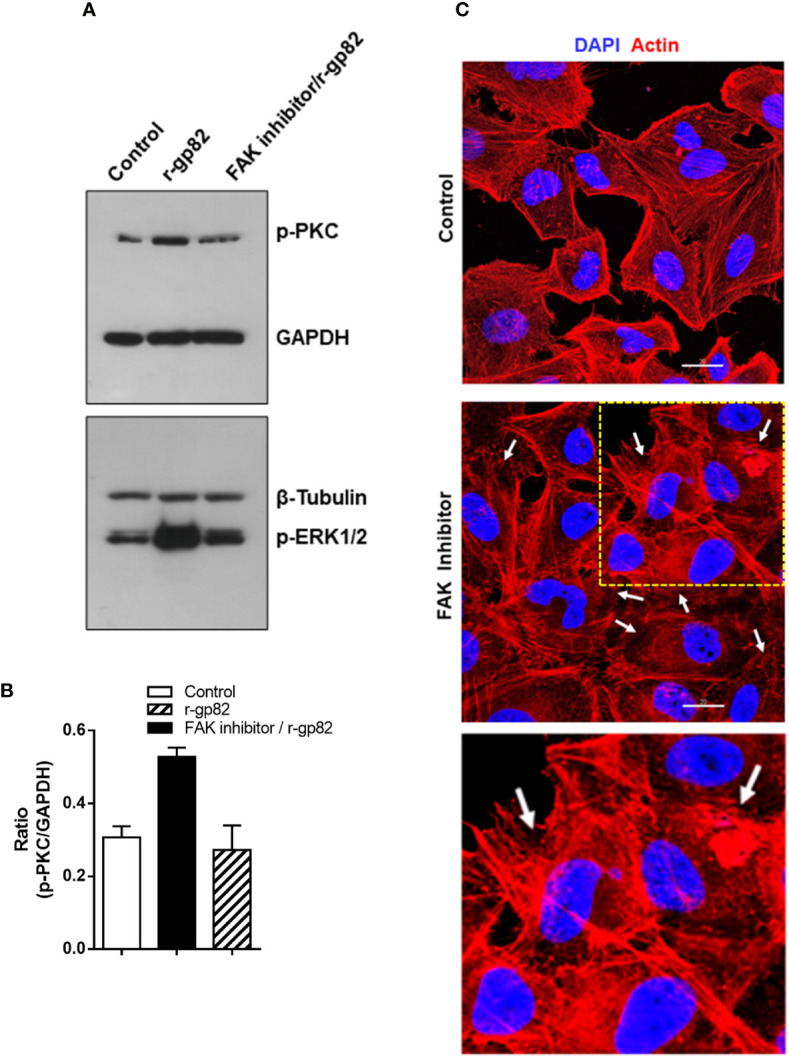
Inhibition of gp82-induced activation of host cell PKC and ERK1/2 by FAK inhibitor. **(A)** HeLa cells, untreated or pretreated with FAK inhibitor, were incubated for 30 min in absence or in the presence of r-gp82, and analyzed by Western blotting for detection of phosphorylated PKC and ERK1/2. Note that treatment with FAK inhibitor counteracted the gp82-induced activation of PKC and ERK1/2. **(B)** Shown is the ratio of p-PKC/GAPDH. Values are the means ± SD of duplicate assays. **(C)** HeLa cells, untreated or treated with FAK inhibitor, were processed for visualization of actin cytoskeleton (red) and nucleus (blue). Scale bar = 20 µm. Note the extensively disorganized actin cytoskeleton in cells treated with FAK inhibitor (white arrows). To facilitate visualization, a magnified image from the framed area (square) is also shown.

A previous study showed that treatment of HeLa cells with specific FAK inhibitor PF573228 results in ERK1/2 dephosphorylation, alteration in the actin cytoskeleton architecture and higher resistance to gp82-mediated MT invasion (Onofre et al., 2019). Here we examined whether FAK inhibitor affected gp82-induced PKC phosphorylation. HeLa cells were either untreated or treated for 45 min with 40 µg/ml FAK inhibitor in serum-free medium. Untreated and FAK inhibitor-treated cells were then incubated for 30 min with r-gp82 at 10 µg/ml, and processed for western blotting analysis. FAK inhibitor blocked PKC and ERK1/2 activation induced by r-gp82 (**Figure 6A**). PKC activation by r-gp82 and inhibition by FAK inhibitor was confirmed by densitometry in a repeated assay (**Figure 6B**). Actin cytoskeleton disorganization was detectable upon 30 min incubation of HeLa cells with FAK inhibitor (**Figure 6C**). A more extensive F-actin disarrangement was observed in cells treated with FAK inhibitor (**Figure 6C**) than in cells incubated with r-gp82 (**Figure 5D**).

### Phospholipase C Inhibitor Blocks Gp82-Induced Protein Kinase C Activation and Lysosome Mobilization and Inhibits Metacyclic Trypomastigote Invasion

**Figure 7:**
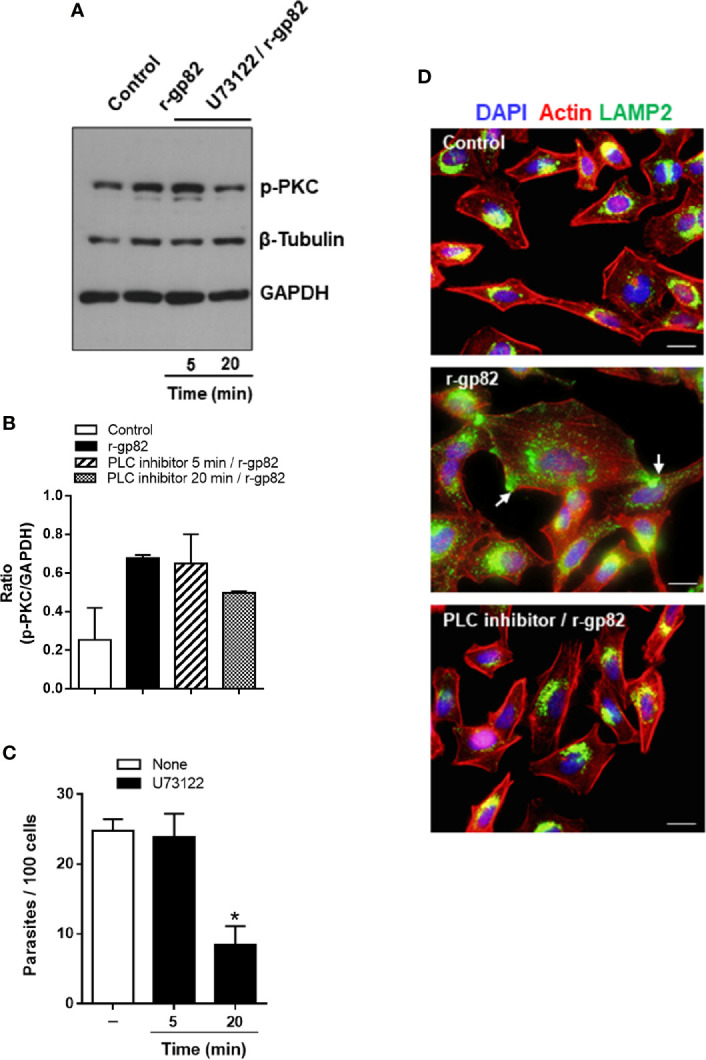
Blockage of gp82-induced activation of host cell PKC by PLC inhibitor. **(A)** HeLa cells, untreated or pretreated with PLC inhibitor, for the indicated time, were incubated for 30 min in absence or in the presence of r-gp82, and analyzed by western blotting for detection of phosphorylated PKC. Note that treatment of cells with PLC inhibitor for 20 min impaired the gp82-induced activation of PKC. **(B)** The ratio of p-PKC/GAPDH is shown. Values are the means ± of two assays. **(C)** HeLa cells, untreated or pretreated with PLC inhibitor for the indicated time, were incubated for 1 h with MT, and the internalized parasites was quantified. Values are the means ± SD of five independent assays performed in duplicate. MT internalization was significantly inhibited in cells pretreated with PLC inhibitor for 20 min (*P < 0.0001). **(D)** HeLa cells, untreated or pretreated with U73122 for 20 min, were incubated with r-gp82 for 30 min and processed for immunofluorescence microscopy. The cortical F-actin structure and the lysosome localization were similar in untreated control and in cells pretreated with U73122 min and incubated with r-gp82.

The PKC isoform (α and/or β) activated in HeLa cells upon interaction with MT or r-gp82 belongs to the group of classical PKCs that are activated by Ca^2+^ (Huang et al., 1986) and diacylglycerol (DAG) (Nishizuka, 1986). This suggested that PLC, which generates DAG and inositol 1,4,5-triphosphate (IP3) that releases Ca^2+^ from intracellular reservoirs (Streb et al., 1983) was implicated in PKC activation. To determine the involvement of PLC in gp82-induced PKC activation, HeLa cells were incubated for 5 or 20 min with specific PLC inhibitor U73122, at 10 µM, a concentration used for treatment of different cell types (Berven and Barritt, 1995; Muto et al., 1997). After removal of the drug, the cells were incubated with 20 μg/ml r-gp82 for 30 min. Extracts of untreated and U73122-treated cells were prepared and analyzed by western blotting for detection of phosphorylated PKC. Treatment of cells with PLC inhibitor for 20 min blocked gp82-induced PKC activation, as visualized in the western blot (**Figure 7A**) and confirmed in a repeated assay, as shown by densitometry analysis (**Figure 7B**). Next, the effect of U73122 on MT invasion was examined. HeLa cells, untreated or pretreated with 10 µM U73122 for 5 or 20 min, were incubated with MT for 1 h and processed for internalized parasite quantification. Pretreatment of HeLa cells for 20 min, but not for 5 min, significantly increased the resistance to MT invasion (**Figure 7C**). An experiment was also performed to determine whether pretreatment of cells with U73122 interfered with the gp82 activity in inducing lysosome spreading, provided that requirement of PLC on lysosome exocytosis has been reported (Andrei et al., 2004). HeLa cells were pretreated with U73122 for 20 min, and then were incubated with r-gp82 for 30 min, and processed for immunofluorescence microscopy. In cells pretreated with PLC inhibitor, the lysosome spreading induced by r-gp82 was impaired, and the perinuclear lysosome localization was similar to that observed in untreated cells (**Figure 7D**).

### Phorbol Ester PMA Activates Protein Kinase C, Disorganizes Actin Cytoskeleton, and Inhibits Lysosome Mobilization

**Figure 8:**
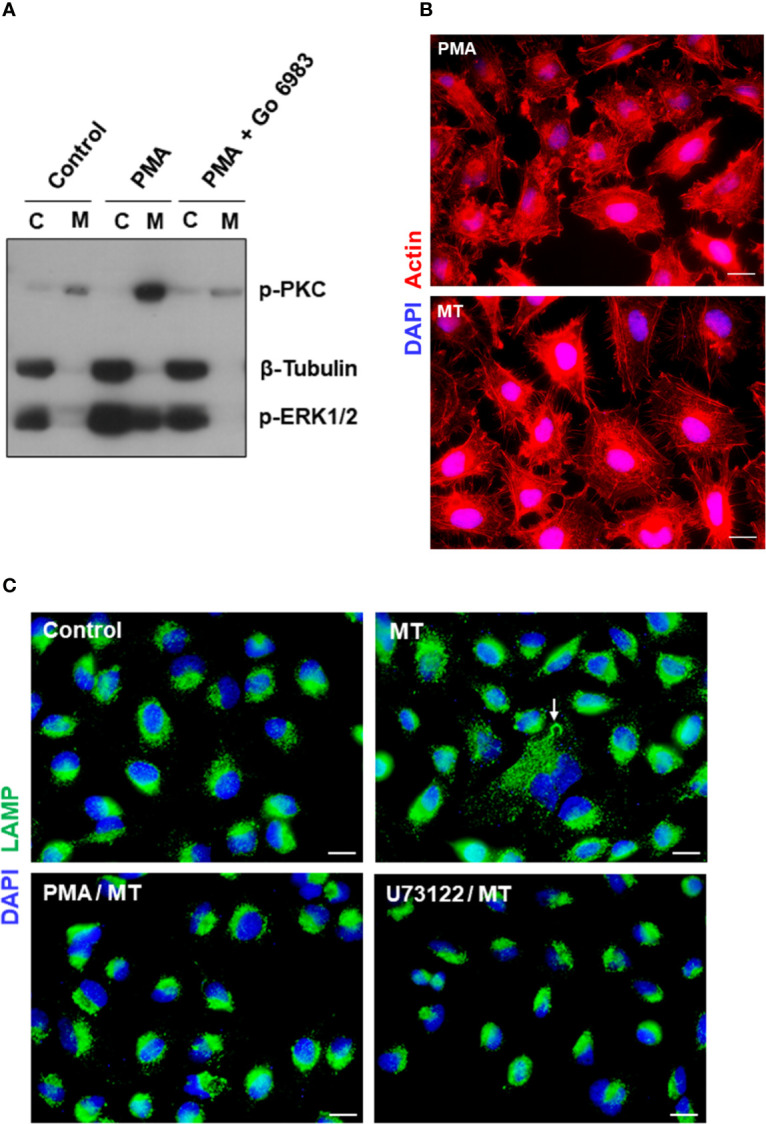
Effect of PKC activator PMA on cytoskeleton disruption and MT-induced lysosome mobilization. **(A)** HeLa cells were untreated or treated with PMA, in absence or in the presence of PKC inhibitor Go 6983, and the membrane (M) and cytosolic (C) fractions were analyzed for detection of phoshorylation levels of PKC and ERK1/2. **(B)** HeLa cells, untreated or treated with PMA, were incubated with MT for 30 min and processed for immunofluorescence microscopy to visualize actin cytoskeleton (red) and nucleus (blue). Scale bar = 10 µm. **(C)** HeLa cells, untreated or treated with PMA or PLC inhibitor U73122, were incubated with MT for 30 min and processed for immunofluorescence microscopy to visualize lysosomes (green). In untreated cells that interacted with MT, lysosome spreading induced by the parasite (white arrow) can be seen. Scale bar = 10 µm.

We have found in previous studies that treatment of HeLa cells with phorbol ester PMA inhibits MT invasion by blocking the spreading of lysosomes and exocytosis (Martins et al., 2011). PMA, an activator of PKC, has been shown to inhibit diverse cell processes, such as angiotensin-induced activation of PLC (Brock et al., 1985), alfa 1-adrenergic responses, including the increase in free cytosolic Ca^2+^ and release of IP3 (Lynch et al., 1985), phosphoinositide hydrolysis and cytosolic Ca^2+^ rise induced by muscarinic receptor activation (Vicentini et al., 1985). To determine the effect of PMA on HeLa cells that could reduce the susceptibility to MT invasion, a set of experiments were carried out. First, HeLa cells were untreated or treated with 100 nM PMA for 30 min, in absence or in the presence of a broad spectrum PKC inhibitor Go 6983 at 2 nM, and the membrane and cytosolic fractions were analyzed for detection of phoshorylation levels of PKC and ERK1/2, using β-tubulin as fractionation and loading control. Highly activated PKC was detected in the membrane fraction, and ERK1/2 in the cytosolic fraction, of PMA-treated cells (**Figure 8A**). Next, we compared the actin cytoskeletal structure in untreated and PMA-treated cells, upon 30 min interaction with MT. PMA-treated cells exhibited a highly disorganized actin cytoskeleton, with an appearance distinct from that induced by MT (**Figure 8B**). In cells incubated with MT, the actin stress fibers are more preserved and membrane ruffles, such as seen in PMA-treated cells, are not visualized. As regards lysosome spreading, pretreatment with PMA or PLC inhibitor U73122 rendered the HeLa cells unresponsive to MT-induced mobilization of lysosomes, which remained concentrated perinuclearly (**Figure 8C**).

## Discussion

Our results have indicated that interaction of MT gp82 with its host cell receptor LAMP2 induces the signaling pathway that involves the activation of PKC and the downstream ERK1/2. The process of gp82-mediated MT invasion resembles therefore the stimulation of different cell types by diverse factors. For instance, PKC was required for activation of ERK1/2 in mycobacterial infection of macrophages (Yadav et al., 2006) or upon proinflammatory stimulation by basic calcium phosphate crystals (Nadra et al., 2005).

The MT gp82 sequence involved in host cell attachment was identified twenty years ago (Manque et al., 2000), but only recently the target cell receptor for gp82 was identified. LAMP2, which is expressed at low levels in the plasma membrane of HeLa cells, was found to function as receptor for gp82 (Rodrigues et al., 2019). Here we mapped the LAMP2 sequence predicted to bind gp82. The synthetic peptide based on that sequence impaired the binding of r-gp82 to HeLa cells, as well as MT internalization, and blocked the gp82-induced PKC and ERK1/2 activation.

We have found that the PKC activated in HeLa cells by MT or r-gp82 belongs to the group of Ca^2+^-activated PKCs. This is in agreement with the requirement of Ca^2+^ in the process of MT invasion (Dorta et al., 1995; Ruiz et al., 1998) and with the fact that the gp82-induced disruption of target cell actin cytoskeleton, which is associated with lysosome spreading (Martins et al., 2011), is Ca^2+^-dependent (Cortez et al., 2006). The source of Ca^2+^ is presumably IP3-sensitive stores, provided the gp82-induced PKC activation, as well as MT invasion, was impaired by treatment of HeLa cells with PLC inhibitor. We do not know in which way gp82-LAMP2 interaction could activate PLC. As reviewed in (Kadamur and Ross, 2013), of six mammalian PLC families, each respond to its own spectrum of activators that includes heterotrimeric G protein subunits, protein tyrosine kinases, small G proteins, Ca^2+^, and phospholipids. One possibility is that gp82-LAMP2 interaction might induce PLC activation, through one of the mentioned activators. Ca^2+^-dependent PKC translocates from cytosol to plasma membrane upon activation by diverse stimuli (Kraft and Anderson, 1983; Hirota et al., 1985). Accordingly, activated PKC was found mostly in the membrane fraction of HeLa cells upon interaction with MT or r-gp82.

The association of PKC/ERK1/2 signaling with actin cytoskeleton organization, described in different cell types (Nurminsky et al., 2007; Wang and Hatton, 2007), was also observed in cells treated with phorbol ester PMA. However, PMA-induced extensive disassembly of actin stress fibers, with concomitant appearance of membrane ruffles, had the effect of inhibiting the lysosome mobilization induced by MT, what is compatible with previous findings that pretreatment of cells with PMA inhibits MT invasion (Cortez et al., 2006; Martins et al., 2011). As the morphology of the cytoskeleton is regulated by a large number of components and can be modified by many exogenous stimuli (Larsson, 2006), we presume that different factors trigger distinct signaling pathways, which may have in common the activation of PKC and ERK1/2, but lead to distinct F-actin rearrangements. In HeLa cells treated with FAK inhibitor, which blocked the gp82-induced PKC/ERK1/2 activation, the profile of rearrangement of F-actin differed from that induced by gp82/MT or PMA. The actin reorganization promoted by FAK inhibitor or PMA may have an adverse effect either on MT invasion process (Onofre et al., 2019) and/or in the retention of parasites. Studies with TCT have reported that transient depolymerization of the cortical actin cytoskeleton facilitates parasite invasion, but actin reassembly is required for the formation of a parasitophorous vacuole with lysosomal properties, in order to prevent parasites from exiting host cells (Andrade and Andrews, 2004; Woolsey and Burleigh, 2004).

Although the transient increase in host cell cytosolic Ca^2+^ concentration and lysosome spreading are common features of MT and TCT invasion (Rodríguez et al., 1995; Docampo and Moreno, 1996; Ruiz et al., 1998; Martins et al., 2011), the signaling pathways triggered by these parasite forms in the host cell are distinct. In a study using normal rat kidney cells, it has been suggested that PKC activity is not required for TCT invasion, on the basis that treatment of cells with PKC inhibitors did not impair calcium response or the centripetal F-actin reorganization, and did not affect the efficiency of parasite internalization (Rodríguez et al., 1995). The authors did not test the effect of phorbol esters, which greatly increase the affinity of PKC for Ca^2+^ and enhance the enzyme activity (Castagna et al., 1982). The difference in signaling pathways triggered by MT and TCT in target cells is presumably associated with the fact that, differently from MT, the TCT entry is initiated by plasma membrane invagination (Woolsey et al., 2003). In a lysosome exocytosis- dependent process, TCT induces plasma membrane injury and a rapid form of endocytosis that internalizes membrane lesions (Fernandes et al., 2011).

**Figure 9:**
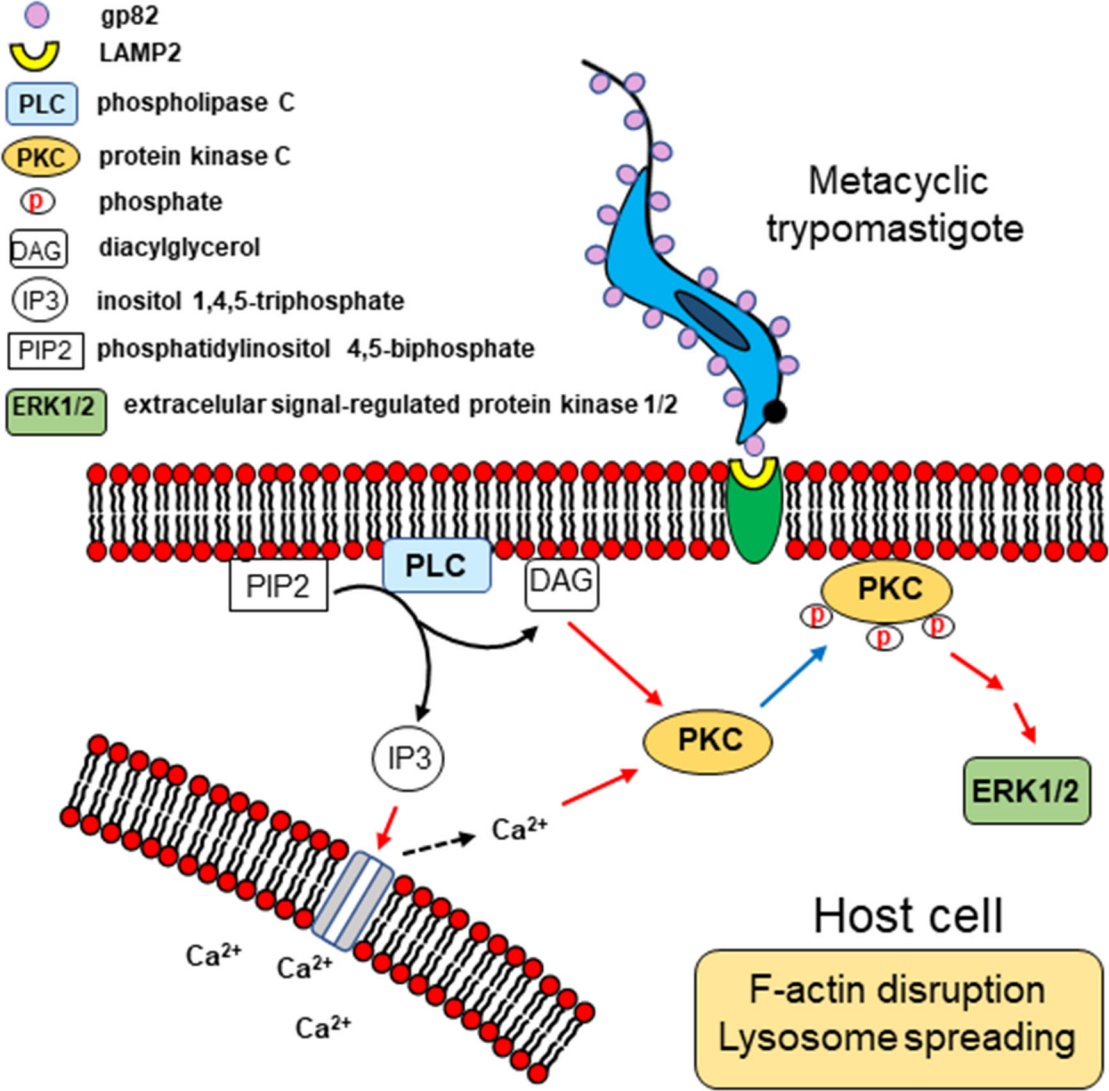
Schematic representation of signaling molecules and pathways possibly activated during *T. cruzi* MT invasion of HeLa cells. Upon MT gp82 interaction with host cell LAMP2, PLC would be activated, generating DAG and IP3. Then Ca^2+^ would be released from IP3-sensitive reservoir. Both Ca^2+^ and DAG activate PKC, which translocates to the plasma membrane, and then follows the downstream ERK1/2 activation. This cascade of events is associated with the actin cytoskeleton disruption and lysosome spreading.

Our results, together with previous findings, provide a picture of the possible mechanism of gp82-mediated host cell invasion by MT, as depicted schematically in **Figure 9**. Recognition of MT gp82 by LAMP2 induces in the host cell the activation of PLC, with generation of DAG and IP3. By acting on IP3-sensitive intracellular stores, IP3 releases Ca^2+^, thus increasing the cytosolic Ca^2+^ concentration. Both Ca^2+^ and DAG activate PKC, which is translocated to the plasma membrane. Following PKC phosphorylation, the downstream ERK1/2 is activated. This chain of events leads to the actin cytoskeleton rearrangement and lysosome spreading, promoting MT internalization.

## Data Availability Statement

The raw data supporting the conclusions of this article will be made available by the authors, without undue reservation.

## Ethics Statement

All procedures conformed to Brazilian National Committee on Ethics Research (CONEP) guidelines, and the study was approved by the ethical committee for animal experimentation of the Universidade Federal de São Paulo (protocol number 9780200918).

## Author Contributions

TO and NY conceived and designed the experiments. TO, JR, and MS performed the experiments, MJ produced the synthetic peptides, and SM generated the recombinant protein. NY wrote the manuscript, and TO helped in writing the part related to modeling. All authors contributed to the article and approved the submitted version.

## Funding

This work was supported by São Paulo Research Foundation (FAPESP) Grant 2016/15000-4, Conselho Nacional de Desenvolvimento Científico e Tecnológico (CNPq) Grant 303825/2015-4 and in part by the Coordenação de Aperfeiçoamento de Pessoal de Nível Superior – Brazil (CAPES) - Finance Code 001.

## Conflict of Interest

The authors declare that the research was conducted in the absence of any commercial or financial relationships that could be construed as a potential conflict of interest.

## Supplementary Material

Supplementary Figure 1 Lack of reactivity of purified r-gp82 with anti-*E. coli* antibody. Purified r-gp82 and *E. coli* extract were analyzed by western blotting, using monoclonal antibody to gp82 and anti-*E. coli* antiserum. Note in the r-gp82 preparation the band detectable by monoclonal antibody to gp82, but no bands corresponding to bacterial components recognized by anti-*E. coli* antiserum.

Supplementary Figure 2 Host cell invasion by *T. cruzi* MT. **(A)** HeLa cells were incubated with MT for 1 h and then stained with Giemsa for internalized parasite counting. Values are the means ± SD of five independent assays performed in duplicate. Shown in **(B)** is a cell harboring one parasite (arrow), surrounded by non-infected cells. Adherent parasites are also seen. Shown in **(C)** are cells harboring one parasite and a binucleated cell with four internalized parasites (arrows). Scale bar = 10 µm.

Supplementary Figure 3 Lysosome-dependent *T. cruzi* MT internalization. HeLa cells were incubated with MT for 30 min and then processed for confocal fluorescence microscopy to visualize lysosomes (green), nucleus (blue), and non-internalized parasites (red). Scale bar = 10 µm. Note the internalized MT with lysosome marker (white arrows) and lysosome accumulation at the cell edges (yellow arrows) in binucleated large cells.

Supplementary Figure 4 Relative positioning of lysosomes upon incubation of cells with r-gp82. HeLa cells treated or not with r-gp82 (**Figure 3B**) were analyzed by plotting green pixels (lysosomes) and blue pixels (nucleus) in a histogram. The lysosomes positioned away from the nucleus were then plotted in a histogram. The peak signal intensity in the presence of r-gp82 is indicated by red arrow.

Supplementary Figure 5 PKC activation induced by gp82-mediated interaction of MT with host cells. The parasites were incubated in absence or in the presence anti-gp82 monoclonal antibody for 30 min and then were seeded onto HeLa cells. After 30 min incubation, the cells that interacted with MT and the control cells that had no contact with parasites were processed for detection of phosphorylated PKC. Anti-gp82 monoclonal antibody reduced the capacity of MT in activating PKC.
