# Supplementary figures and images for: Interaction of Trypanosoma cruzi Gp82 With Host Cell LAMP2 Induces Protein Kinase C Activation and Promotes Invasion
Source: Front Cell Infect Microbiol. 2021 Mar 12;11:627888. doi: 10.3389/fcimb.2021.627888 (PMC7996063; doi:10.3389/fcimb.2021.627888)

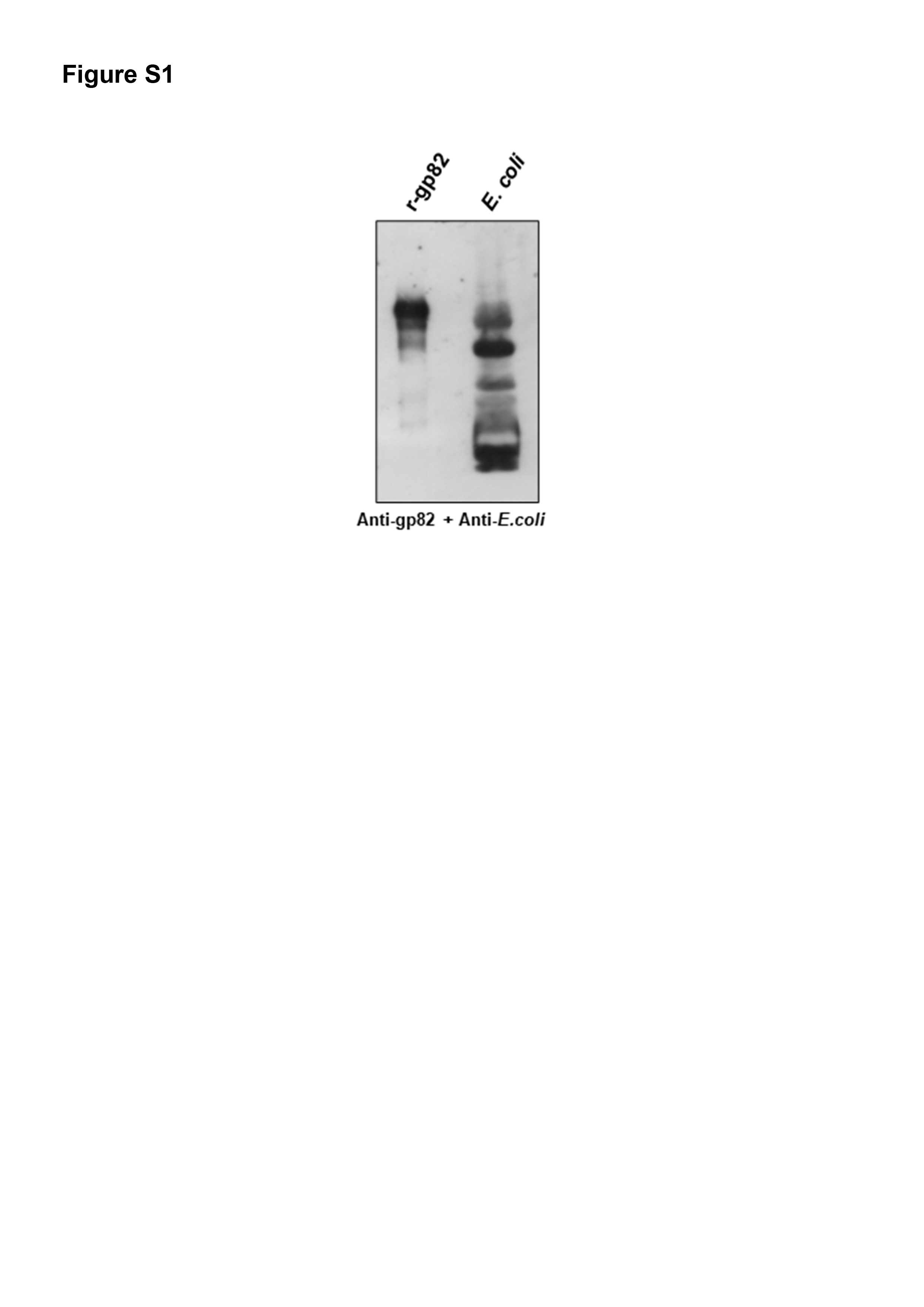

Supplement: Supplementary Figure 1 — Lack of reactivity of purified r-gp82 with anti-E. coli antibody. Purified r-gp82 and E. coli extract were analyzed by western blotting, using monoclonal antibody to gp82 and anti-E. coli antiserum. Note in the r-gp82 preparation the band detectable by monoclonal antibody to gp82, but no bands corresponding to bacterial components recognized by anti-E. coli antiserum. [file Image_1.tif]

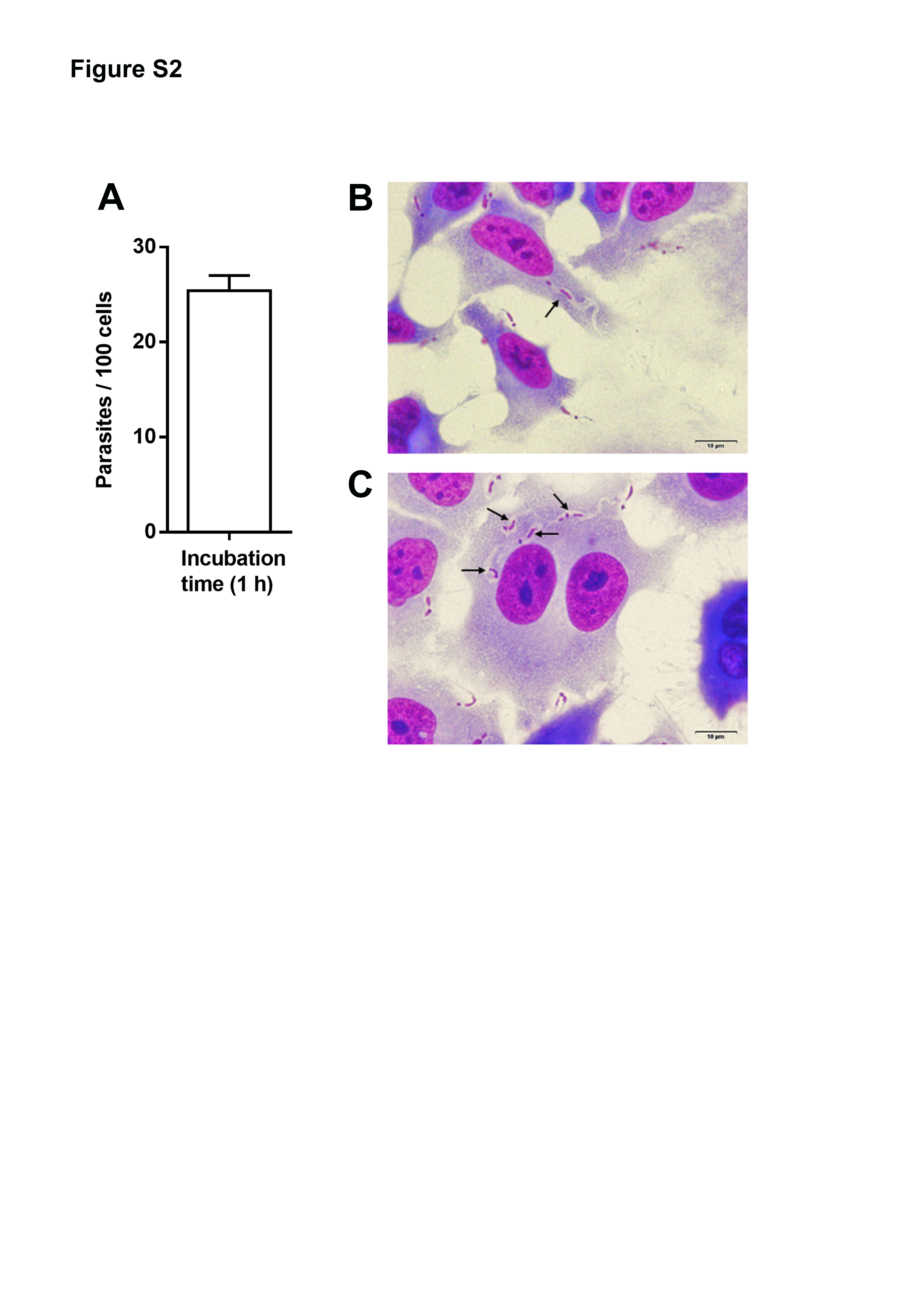

Supplement: Supplementary Figure 2 — Host cell invasion by T. cruzi MT. (A) HeLa cells were incubated with MT for 1 h and then stained with Giemsa for internalized parasite counting. Values are the means ± SD of five independent assays performed in duplicate. Shown in (B) is a cell harboring one parasite (arrow), surrounded by non-infected cells. Adherent parasites are also seen. Shown in (C) are cells harboring one parasite and a binucleated cell with four internalized parasites (arrows). Scale bar = 10 µm. [file Image_2.tif]

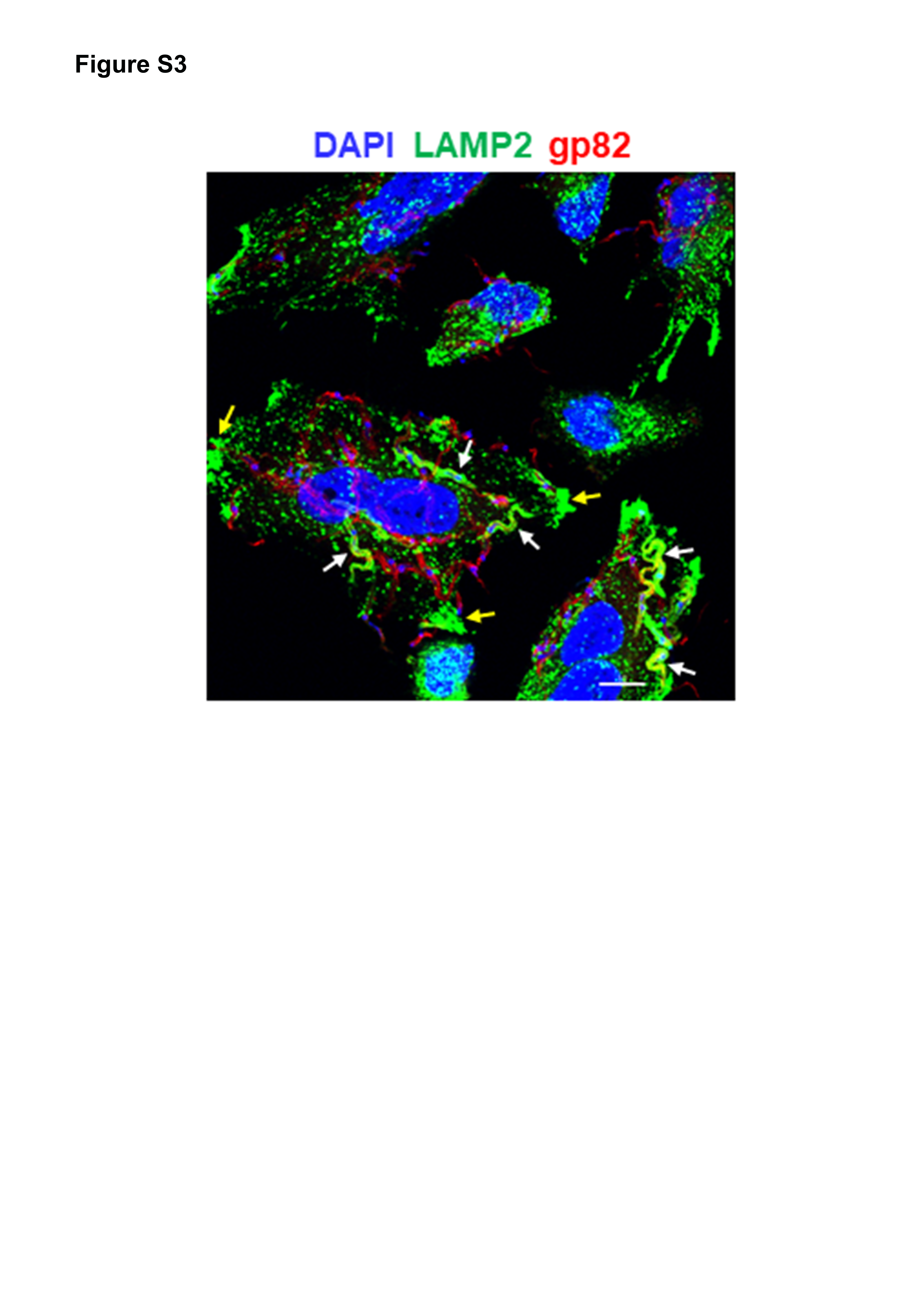

Supplement: Supplementary Figure 3 — Lysosome-dependent T. cruzi MT internalization. HeLa cells were incubated with MT for 30 min and then processed for confocal fluorescence microscopy to visualize lysosomes (green), nucleus (blue), and non-internalized parasites (red). Scale bar = 10 µm. Note the internalized MT with lysosome marker (white arrows) and lysosome accumulation at the cell edges (yellow arrows) in binucleated large cells. [file Image_3.tif]

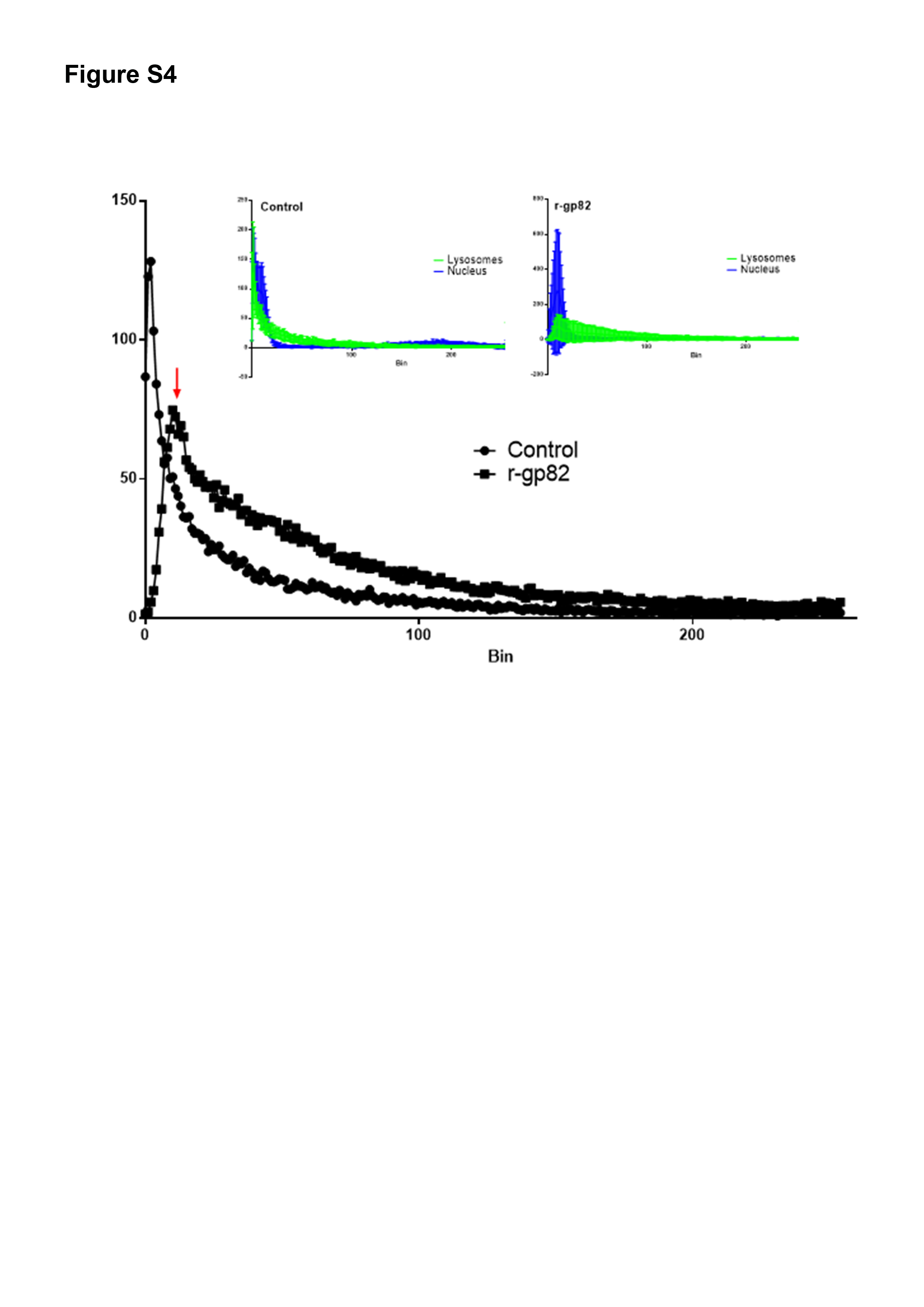

Supplement: Supplementary Figure 4 — Relative positioning of lysosomes upon incubation of cells with r-gp82. HeLa cells treated or not with r-gp82 ( Figure 3B ) were analyzed by plotting green pixels (lysosomes) and blue pixels (nucleus) in a histogram. The lysosomes positioned away from the nucleus were then plotted in a histogram. The peak signal intensity in the presence of r-gp82 is indicated by red arrow. [file Image_4.tif]

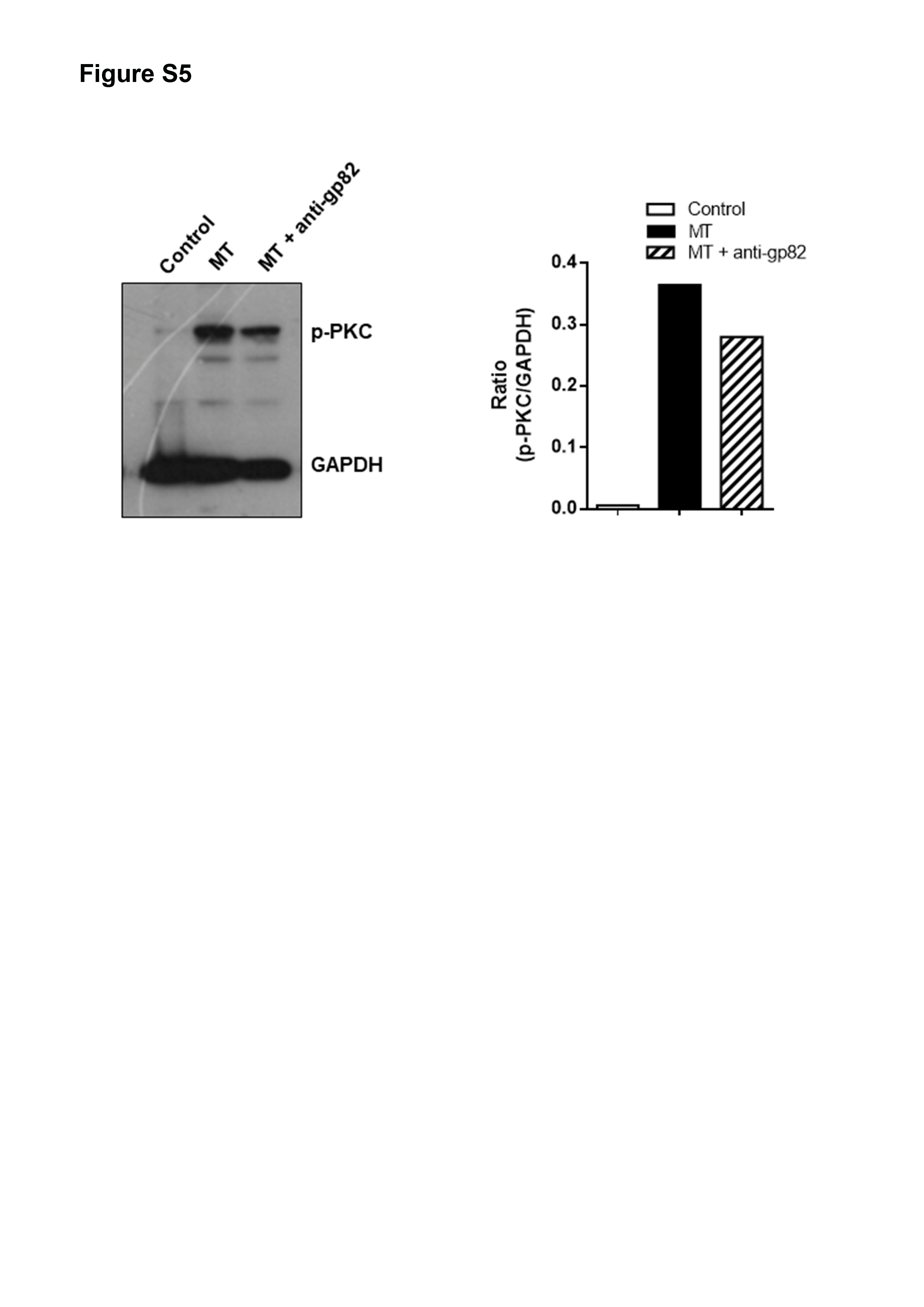

Supplement: Supplementary Figure 5 — PKC activation induced by gp82-mediated interaction of MT with host cells. The parasites were incubated in absence or in the presence anti-gp82 monoclonal antibody for 30 min and then were seeded onto HeLa cells. After 30 min incubation, the cells that interacted with MT and the control cells that had no contact with parasites were processed for detection of phosphorylated PKC. Anti-gp82 monoclonal antibody reduced the capacity of MT in activating PKC. [file Image_5.tif]
